# An oral-origin *Streptococcus salivarius* associated with schizophrenia reshapes intestinal T-cell immunity and promotes IL-17A-dominated inflammation

**DOI:** 10.3389/fimmu.2026.1978713

**Published:** 2026-10-15

**Authors:** Yongyong Kang, Yajuan Fan, Qingyan Ma, Lu Yu, Jing Liu, Yixin Liu, Ziyang Zhou, Huanhuan Zhao, Zai Yang, Yufeng Xun, Min Jia, Binbin Zhao, Xiancang Ma, Wei Wang, Yijie Guo

**Affiliations:** 1 Department of Psychiatry, The First Affiliated Hospital of Xi’an Jiaotong University, Xi’an, China; 2 Genome Institute, Centre for Mathematical Medicine, The First Afffliated Hospital of Xi’an Jiaotong University, Xi’an, China; 3 Center for Brain Science, The First Affiliated Hospital of Xi’an Jiaotong University, Xi’an, China; 4 Center for Translational Medicine, The First Affiliated Hospital of Xi’an Jiaotong University, Xi’an, China

**Keywords:** adaptive immunity, gut-brain axis, oral microbiome, schizophrenia, *Streptococcus salivarius*, Th17/Treg imbalance

## Abstract

**Background:**

Emerging evidence suggests that oral microbial dysbiosis contributes to neuropsychiatric disorders through microbiome–immune interactions. We previously identified an oral-origin *Streptococcus salivarius* enriched in individuals with schizophrenia (SCZ) and demonstrated its ability to become enriched in the intestine after oral administration. However, whether this disease-associated bacterium remodels host adaptive immunity remains unclear. Here, we investigated the effects of *S. salivarius* on intestinal CD4^+^ T-cell homeostasis and inflammatory responses.

**Methods:**

Male C57BL/6 mice were orally administered *S. salivarius* for 14 days with or without prior antibiotic-mediated depletion of resident microbiota. Intestinal enrichment of *S. salivarius* was confirmed by quantitative PCR. Colonic lamina propria mononuclear cells (LPMCs) and mesenteric lymph node (MLN) cells were analyzed using single-cell RNA sequencing and flow cytometry to characterize CD4^+^ T cell responses. Cytokine levels in colon tissues and serum were quantified by ELISA.

**Results:**

Single-cell transcriptomic analysis demonstrated that intestinal enrichment of *S. salivarius* altered Treg-associated transcriptional programs, with reduced Treg-stability signatures and increased Type-17-associated features. Flow cytometry confirmed increased Th17 cells, reduced Treg frequencies, and disruption of intestinal Th17/Treg homeostasis. Similar immune alternations were observed in MLNs, indicating extension of gut-associated immune remodeling beyond the intestinal mucosa. *S. salivarius* exposure was further associated with selective IL-17A in colonic tissues and serum, whereas IL-6, IL-1β, IL-10, and TGF-β remained largely unchanged. Comparable immune changes were observed in both antibiotic-pretreated and conventional SPF mice, indicating that these immunological effects were not restricted to microbiota-depletion condition.

**Discussion:**

These findings identify an SCZ-associated oral-origin *S. salivarius* as a modulator of intestinal adaptive immunity. Intestinal enrichment of *S. salivarius* was associated with coordinated remodeling of Treg-associated transcriptional programs, Th17-biased immune polarization, and an IL-17A-dominated inflammatory response, Together, these results define an intestinal immune mechanism through which a disease-associated oral bacterium may contribute to oral-gut immune interactions and provide experimental support for one immunological component of the oral-gut-brain axis relevant to schizophrenia.

## Graphical Abstract

**Figure:**
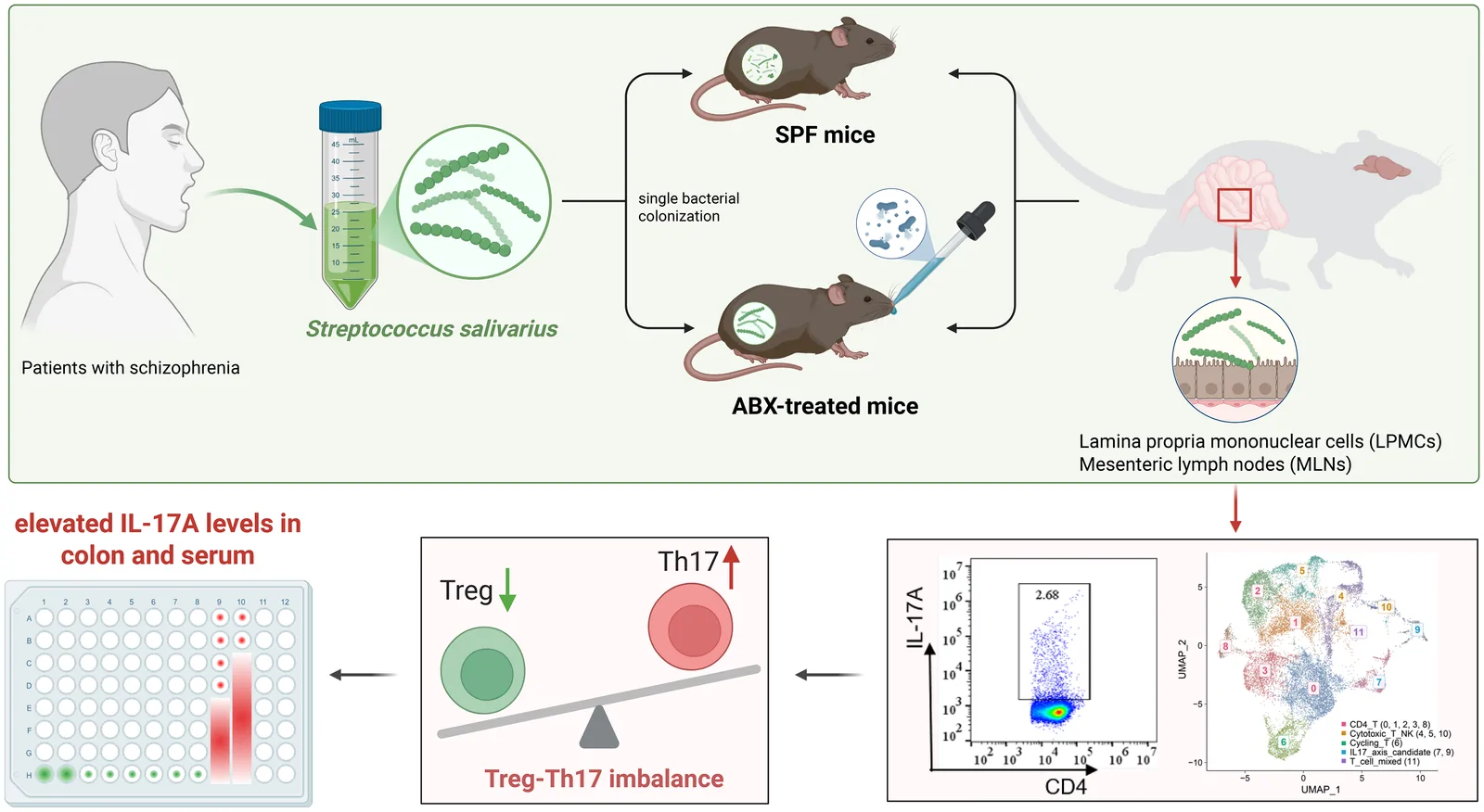

## 1 Introduction

Schizophrenia (SCZ) is a severe and heterogeneous psychiatric disorder affecting approximately 24 million individuals worldwide. Although genetic susceptibility contributes substantially to disease risk, increasing evidence indicates that immune dysregulation and microbial perturbations participate in SCZ pathophysiology. The gut–brain axis has emerged as a critical interface linking microbial alterations with neuropsychiatric disorders, through immune, metabolic, and neural pathways (1, 2). Consistent with this concept, gastrointestinal abnormalities are frequently observed in individuals with SCZ, and gut microbial alterations have been repeatedly reported in clinical cohorts (3–5). However, the specific microbial species and host immune pathways involved in these interactions remain poorly understood.

The oral microbiota represents an important but comparatively underexplored component of the microbiome–immune–brain axis. Although the oral cavity and intestine are separated by distinct anatomical and ecological environments, continuous microbial migration occurs through swallowing, and disruption of host barriers or microbial homeostasis may facilitate the ectopic persistence of oral-derived bacteria in the gastrointestinal tract. Increasing evidence indicates that such ectopic colonization can influence host immunity and contribute to inflammatory diseases (6, 7). For example, oral *Fusobacterium nucleatum* has been implicated in colorectal tumorigenesis through remodeling of the immune microenvironment (8), whereas *Porphyromonas gingivalis* and other oral-associated bacteria has been linked to intestinal inflammation and immune activation (9). Emerging epidemiological evidence further links oral microbial alterations with neuropsychiatric disorders, including associations between oral pathogens (*F. nucleatum* and *P. gingivalis*) and depressive symptoms (10, 11), as well as periodontal disease and schizophrenia risk (12, 13). Nevertheless, whether specific oral-origin bacteria associated with psychiatric disorders can reshape host immunity and thereby contribute to neuroimmune alternations remains unknown.

Our group has systematically investigated microbial and immune alterations associated with SCZ. Through metagenomic analyses, we identified SCZ-associated microbial signatures and demonstrated that gut microbiome alterations can influence SCZ-relevant behavioral phenotypes in experimental models (2). Among these candidates, *Streptococcus salivarius*, an oral-origin bacterium enriched in both fecal and salivary samples from individuals with SCZ, attracted particular attention. Although *S. salivarius* has been implicated as a potential microbial contributor associated with SCZ-related phenotypes in our previous study, whether this bacterium can modulate host immune homeostasis remains unknown. Therefore, we investigated the immunological consequences of intestinal *S. salivarius* enrichment and examined whether this microbial factor establishes a distinct adaptive immune signature.

The intestinal immune system represents a major interface through which microbial signals influence host physiology. Among CD4^+^ T cell subsets, Th17 cells and regulatory T cells (Tregs) maintain immune balance through coordinated inflammatory and regulatory programs. Th17 cells, characterized by RORγt expression and IL-17A production, mediate antimicrobial defense but contribute to pathological inflammation when dysregulated, whereas Foxp3^+^ Tregs preserve immune tolerance and suppress excessive immune activation (14, 15). Disruption of the Th17/Treg balance is a central feature of inflammatory disorders and has also been implicated in SCZ. Increased IL-17A levels, enhanced Th17 responses, and reduced Treg proportions have been reported in patients with SCZ (16–19). Moreover, IL-17A can influence neuroimmune interactions by regulating blood–brain barrier integrity and inflammatory signaling within the central nervous system (20, 21). However, whether a specific SCZ-associated microbial species can promote Th17/Treg dysregulation remains unexplored.

Here, we investigated the immunological consequences of intestinal enrichment of a representative SCZ-associated *S. salivarius* isolate following repeated oral administration. Using single-cell RNA sequencing, flow cytometry, and cytokine profiling, we characterized CD4^+^ T-cell responses in the intestinal lamina propria, mesenteric lymph nodes, and peripheral circulation. We aim to define the intestinal adaptive immune remodeling associated with exposure to this disease-associated bacterium, with particular emphasis on Th17/Treg homeostasis, rather than establish a causal relationship between *S. salivarius* and schizophrenia.

## 2 Materials and methods

### 2.1 Mice

Specific pathogen-free (SPF) wild-type C57BL/6 male mice were obtained from Experimental Animal Center of Xi’an Jiaotong University Medical College. All mice were maintained under SPF conditions at 20–22 °C and 45–65% relative humidity under a 12-h light/12-h dark cycle, with food and water provided *ad libitum*. All animal procedures were approved by the Institutional Animal Care and Use Committee of Xi’an Jiaotong University (Approval No. XJTUAE2024-493).

### 2.2 Bacteria culture and intestinal enrichment models

A representative *S. salivarius* isolate previously obtained from the oral cavity of an individual with SCZ (2). Only this representative SCZ-associated isolate was used throughout the present study, and no healthy donor-derived or reference *S. salivarius* strains were included for comparison. Bacteria were cultured overnight in Brain-Heart infusion Broth (BHI) at 37 °C, washed twice with sterile phosphate-buffered saline (PBS), and resuspended in PBS at a final concentration of 1×10^9^ CFU/ml immediately before administration.

To evaluate the immunological effects of *S. salivarius* under different microbial backgrounds, two complementary intestinal colonization models were established. For the antibiotic-pretreated (ABX) model, mice received an antibiotic cocktail by oral gavage once daily for 7 consecutive days, consisting of vancomycin (100 mg/kg), neomycin (200 mg/kg), metronidazole (200 mg/kg), and amphotericin B (2 mg/kg), while ampicillin (2 g/L) was supplied in the drinking water. Following antibiotic withdrawal, mice received regular drinking water for 3 days to allow antibiotic clearance. Substantial depletion of cultivable intestinal bacteria was confirmed by aerobic and anaerobic culture of fecal pellets on BHI agar.

For both the ABX and conventional SPF models, mice were randomly assigned to receive either *S. salivarius* (200 μl, 1 × 10^9^ CFU/ml) or an equivalent volume of PBS by oral gavage once daily for 14 consecutive days, generating four experimental groups: antibiotic-pretreated control (ABX-Ctrl), antibiotic-pretreated *S. salivarius* (ABX-SS), SPF control (SPF-Ctrl), and SPF *S. salivarius* (SPF-SS). Intestinal enrichment of *S. salivarius* was assessed at the experimental endpoint by quantitative PCR targeting *S. salivarius*-specific 16S rRNA gene sequences. Because bacterial abundance was measured only at the end of the administration period, these analyses were designed to confirm intestinal enrichment during treatment rather than long-term persistence after bacterial withdrawal. It should be noted that *S. salivarius* was detectable at low abundance in the SPF control group because it is a naturally occurring commensal bacterium. Bacterial abundance was quantified by relative qPCR, with the control group normalized to a value of 1; therefore, the control group represents the baseline endogenous abundance rather than the absence of *S. salivarius*.

### 2.3 Isolation of colonic lamina propria mononuclear cells

Colons were collected and flushed with ice-cold PBS, adherent adipose tissue and Peyer’s patches were carefully removed, and the tissues were cut into small fragments. To remove epithelial cells, tissues fragments were subjected to two consecutive incubations for 20 min at 37 °C with shaking at 250 rpm in Ca²^+^/Mg²^+^-free Hank’s balanced salt solution (HBSS) containing 5 mM EDTA, 1 mM dithiothreitol (DTT), and 5% fetal bovine serum (FBS).

The remaining tissue fragments were subsequently digested for 30 min at 37 °C in RPMI-1640 supplemented with 10% FBS, 1 mg/mL collagenase type I (Sigma-Aldrich), and 0.1 mg/mL DNase I (Roche). The resulting cell suspensions were passed through a 100-μm cell strainer and centrifuged. Cell pellets were resuspended in 40% Percoll (Solarbio), carefully layered over 70% Percoll, and centrifuged at 500 × g for 30 min at room temperature without brake. Lamina propria mononuclear cells (LPMCs) at the 40%/70% Percoll interface were collected, washed, and immediately used for downstream applications.

### 2.4 Preparation of single-cell suspensions from mesenteric lymph nodes

Mesenteric lymph nodes (MLNs) were collected and mechanically dissociated to generate single-cell suspensions. Cell suspensions were passed through 70-μm cell strainers and washed twice in RPMI-1640 containing 2% FBS. Cell numbers and viability were determined by trypan blue exclusion before downstream analyses.

### 2.5 Single-cell sample preparation, library construction and sequencing

Single-cell RNA sequencing (scRNA-seq) was performed using colonic LPMCs isolated from ABX-Ctrl and ABX-SS mice. Five mice were included in each group, following 14 days of oral administration with *S. salivarius* or PBS. Colonic LPMCs were isolated as described above. Cell concentration and viability were assessed before library preparation, and only high-quality single-cell suspensions were used for sequencing.

Single-cell suspensions from individual mice were processed independently for library construction using the Chromium Single Cell 5’ Gene Expression platform (10x Genomics) according to the manufacturer’ s instructions. Briefly, single cells were encapsulated with barcoded gel beads in emulsion, followed by reverse transcription, cDNA amplification, and construction of 5’ gene-expression libraries. Libraries were sequenced on a high-throughput Illumina sequencing platform using paired-end sequencing.

### 2.6 scRNA-seq data processing and analysis

Raw sequencing data were processed using Cell Ranger v8.0.1 (10x Genomics) for read alignment, transcript quantification, and generation of filtered feature-barcode matrices. Downstream analyses were performed using Seurat v4.4.0 in R v4.3.3.

Genes detected in fewer than three cells were excluded. Cells with 200–7,500 detected features, at least 500 total RNA counts, and <20% mitochondrial transcripts were retained. After quality-control filtering, 55,494 cells across all samples were included in subsequent analyses. Data normalization and variance stabilization were performed using SCTransform, followed by reciprocal principal component analysis (RPCA)-based integration of individual samples. Principal component analysis, UMAP dimensionality reduction, and graph-based clustering were then performed on the integrated dataset.

Major cell populations were annotated according to established lineage-specific markers. T cells were subsequently extracted and reclustered to resolve CD4^+^ T-cell subpopulations, including regulatory T cells (Tregs), which were identified based on canonical markers such as *Foxp3*, *Il2ra*, *Ctla4*, and *Tigit*.

Treg transcriptional states were assessed using UCell v2.6.2. Four predefined transcriptional programs were evaluated: Treg stability-associated (*Foxp3*, *Il2ra*, *Ctla4*, *Ikzf2*, *Tigit*, *Tnfrsf18*, *Tnfrsf4*, and *Il10*), Treg suppressive-function-associated (*Ctla4*, *Il10*, *Entpd1*, *Tigit*, *Itgb8*, and *Icos*), Type-17-poised (*Rorc*, *Rora*, *Ccr6*, *Il23r*, *Il1r1*, *Ahr*, *Maf*, *Batf*, *Irf4*, and *Stat3*), and Type-17 effector programs (*Il17a*, *Il17f*, *Il22*, *Ccl20*, and *Csf2*).

For sample-level comparisons, UCell scores were averaged within each biological sample, and individual mice were treated as biological replicates. Treg-low and Type-17-poised states were defined using empirical thresholds derived from the distribution of corresponding UCell scores in control samples. Cells meeting the predefined low Treg-stability and/or high Type-17-poised criteria were quantified within each sample. Marker-positive cells were defined by detectable expression of the corresponding gene in the raw RNA count matrix.

Transcriptome-wide pseudobulk differential-expression analysis was additionally performed for cluster 8 Treg cells. Raw gene counts from cells belonging to cluster 8 were aggregated at the biological-sample level, with five control and five SS-treated mice included as independent biological replicates. Genes with insufficient expression were filtered using filterByExpr, followed by trimmed mean of M-values (TMM) normalization. Differential expression between the SS and control groups was assessed using the robust quasi-likelihood framework implemented in edgeR. Benjamini–Hochberg (BH) correction was applied to account for multiple testing, and genes with a BH-adjusted false discovery rate (FDR) < 0.05 were considered statistically significant.

To identify coordinated pathway-level transcriptional changes independently of the predefined Treg-associated gene signatures, preranked gene set enrichment analysis (GSEA) was performed using the fgsea R package. All genes tested in the edgeR analysis were ranked according to their signed differential-expression statistics without applying a gene-level significance cutoff. Enrichment was evaluated separately against mouse MSigDB Gene Ontology (GO) Biological Process and Reactome gene-set collections. BH correction was applied within each gene-set collection, and pathways with BH FDR < 0.05 were considered significantly enriched. For visualization, the top 20 pathways within each collection were ranked by BH FDR and subsequently by the absolute normalized enrichment score (|NES|). Positive NES values indicate enrichment in the SS group, whereas negative NES values indicate enrichment in the control group.

### 2.7 Flow cytometry

Colonic LPMCs and MLN single-cell suspensions were used for flow cytometric analysis. Approximately 1 × 10^6^ cells per sample were incubated with anti-mouse CD16/32 antibody (5 μg/ml) for 10 min at 4 °C to block Fc receptors, followed by staining with fluorochrome-conjugated antibodies against CD45 and CD4 (BioLegend) for 30 min at 4 °C (22, 23).

For intracellular IL-17A detection, cells were stimulated with 50 ng/ml phorbol 12-myristate 13-acetate (PMA), 1 μg/ml ionomycin, and 5 μg/ml brefeldin A (BD Biosciences) for 4 h at 37 °C. Following surface staining, cells were fixed and permeabilized using the Cytofix/Cytoperm kit (BD Biosciences) and subsequently stained with an anti-IL-17A antibody. Th17 cells were identified as live CD45^+^CD4^+^IL-17A^+^ cells.

For Treg cells analysis, surface-stained cells were fixed and permeabilized using the True-Nuclear™ Transcription Factor Buffer Set (BioLegend) and stained intracellularly with an anti-Foxp3 antibody. Tregs were identified as live CD45^+^CD4^+^Foxp3^+^ cells. Dead cells were excluded using Zombie NIR™ viability dye (BioLegend).

Data were acquired using a BD LSRFortessa™ flow cytometer and analyzed with FlowJo v10 (BD Biosciences). Fluorescence compensation was performed using single-stained UltraComp eBeads™ (Invitrogen).

### 2.8 Cytokine quantification by ELISA

Cleaned colonic tissues were weighed and homogenized in ice-cold PBS containing protease inhibitor cocktail (Roche) at a ratio of 1 mL PBS per 100 mg tissue using a Precellys 24 homogenizer. Homogenates were clarified by centrifugation at 12,000 × g for 10 min at 4 °C, and supernatants were collected for cytokine measurements.

Peripheral blood was allowed to clot at room temperature for 30 min and then centrifuged at 1,500 × g for 10 min at 4 °C. Serum was collected and stored at −80 °C until analysis.

Concentrations of IL-17A, IL-6, IL-1β, IL-10, and TGF-β were measured using mouse Ready-SET-Go! ELISA kits (Invitrogen™, Thermo Fisher Scientific) according to the manufacturer’s instructions. Cytokine concentrations in colonic tissue homogenates were normalized to tissue wet weight and expressed as pg/mg tissue. Absorbance was measured at 450 nm using a SpectraMax iD5 plate reader (Molecular Devices).

### 2.9 Statistical analyses

Statistical analyses of animal-based experimental data were performed using GraphPad Prism version 8 (GraphPad Software Inc.), whereas scRNA-seq-derived statistical analyses were performed in R as described above. Individual mice were considered biological replicates for animal-based experiments and sample-level analyses derived from scRNA-seq data. Statistical tests used for individual comparisons are specified in the corresponding figure legends. All statistical tests were two-sided unless otherwise indicated, and *P* < 0.05 was considered statistically significant. For transcriptome-wide differential-expression and pathway-enrichment analyses, multiple-testing correction was performed using the Benjamini–Hochberg procedure, with BH FDR < 0.05 defining statistical significance. To evaluate the independent and interactive effects of intestinal microbiota status and *S. salivarius* treatment, data from the ABX and SPF models were additionally analyzed using two-way ANOVA, with microbiota status (ABX vs. SPF) and bacterial treatment (Ctrl vs. SS) included as fixed factors together with their interaction term.

## 3 Results

### 3.1 Validation of intestinal enrichment of *S. salivarius* during the treatment period

To investigate the effects of repeated oral *S. salivarius* administration and intestinal enrichment on host adaptive immunity, we established two complementary mouse models consisting of ABX and SPF mice (**Figure 1A**). In both models, mice received daily oral gavage of either *S. salivarius* or vehicle control for 14 consecutive days, generating four experimental groups: ABX-Ctrl, ABX-SS, SPF-Ctrl, and SPF-SS.

**Figure 1:**
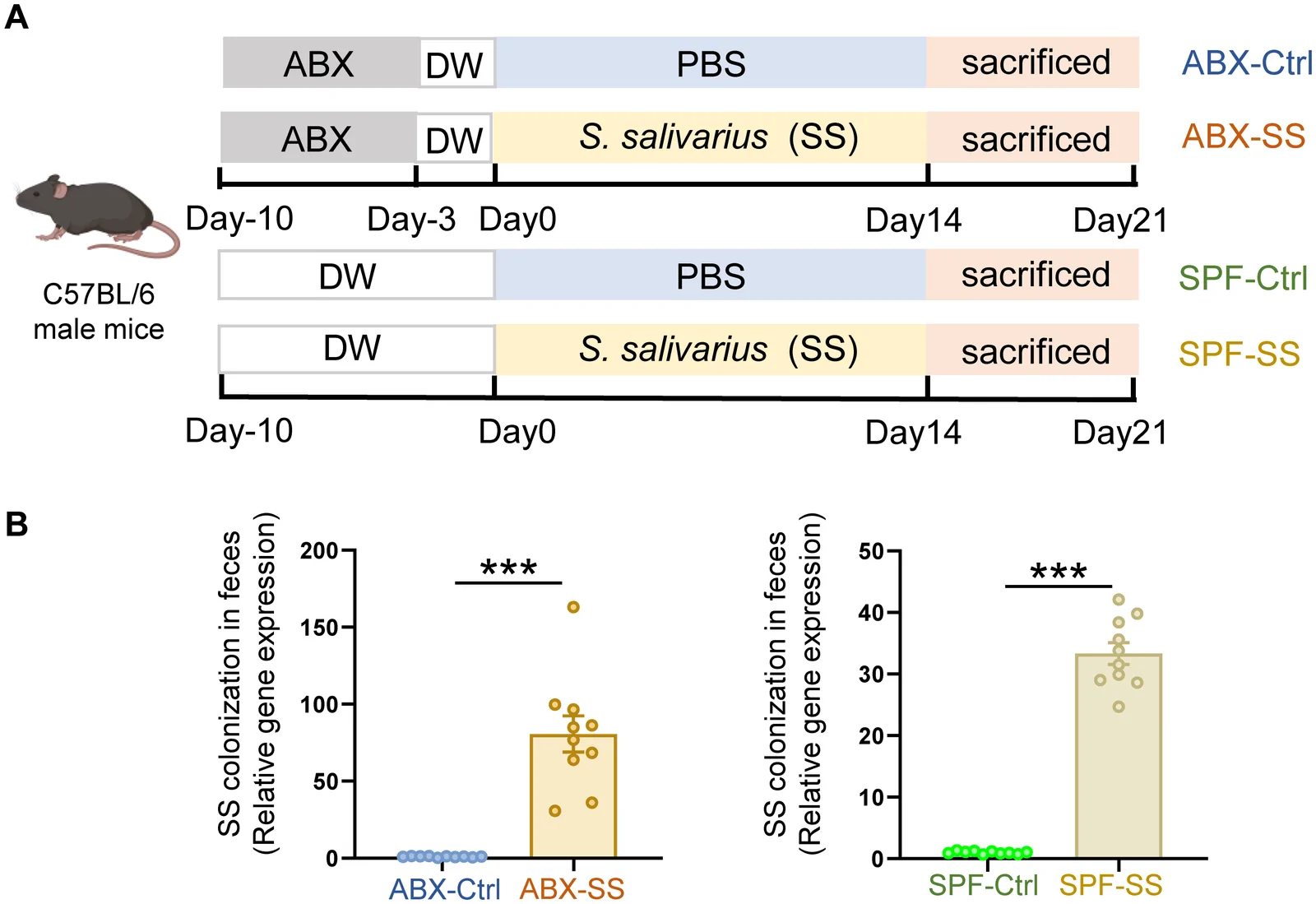
Experimental design and intestinal enrichment of *Streptococcus salivarius* following repeated oral administration. **(A)** Schematic overview of the experimental design for antibiotic-pretreated (ABX) and conventional specific pathogen-free (SPF) mouse models. Male C57BL/6 mice were assigned to four groups: ABX-Ctrl, ABX-SS, SPF-Ctrl, and SPF-SS. Mice in the ABX groups received an antibiotic cocktail from day −10 to day −3 to transiently reduce colonization resistance, followed by regular drinking water from day −3 to day 0 to allow antibiotic washout. From day 0 to day 14, mice were orally gavaged once daily with live *S. salivarius* or phosphate buffered saline (PBS). SPF mice received the same bacterial or vehicle treatment without prior antibiotic treatment. All samples were collected on day 21. **(B)** Relative fecal abundance of *S. salivarius* at the experimental endpoint, determined by quantitative PCR (qPCR). Relative abundance was normalized to the corresponding control group (ABX-Ctrl or SPF-Ctrl), which was set to 1. As *S. salivarius* is a naturally occurring commensal bacterium, low endogenous levels were detectable in SPF-Ctrl mice. Oral administration significantly increased the intestinal abundance of *S. salivarius* in both antibiotic-pretreated and conventional mice compared with their respective control groups. Data are presented as mean ± SEM. Statistical significance was determined using a two-sided Mann–Whitney U test. ***P < 0.001.

Successful intestinal enrichment of *S. salivarius* during the bacterial administration period was confirmed by quantitative PCR targeting species-specific 16S rRNA gene sequences in fecal samples collected at the experimental endpoint. Compared with ABX-Ctrl mice, ABX-SS mice exhibited a marked increase in fecal *S. salivarius* abundance (**Figure 1B**, *P* < 0.001), confirming marked intestinal enrichment following repeated oral administration. Similar enrichment was also detected in SPF mice following *S. salivarius* administration (**Figure 1B**, *P* < 0.001). These complementary experimental models were subsequently used to investigate the immunological consequences of intestinal *S. salivarius* exposure.

### 3.2 Single-cell transcriptomics reveals altered Treg-associated programs and Type-17-associated transcriptional features following *S. salivarius* exposure

To define how *S. salivarius* alters intestinal T cell states, we performed scRNA-seq of colonic immune cells and characterized transcriptional changes within the Treg compartment. Unsupervised clustering of the single-cell dataset identified major immune cell populations, followed by re-clustering of T cells into 12 distinct subpopulations, including CD4^+^ T cells, cytotoxic T/NK cells, cycling T cells, IL-17-associated populations, and mixed T cell subsets (**Figures 2A, B**). Cluster 8 was annotated as Tregs based on canonical marker expression and was selected for subsequent functional analysis.

**Figure 2:**
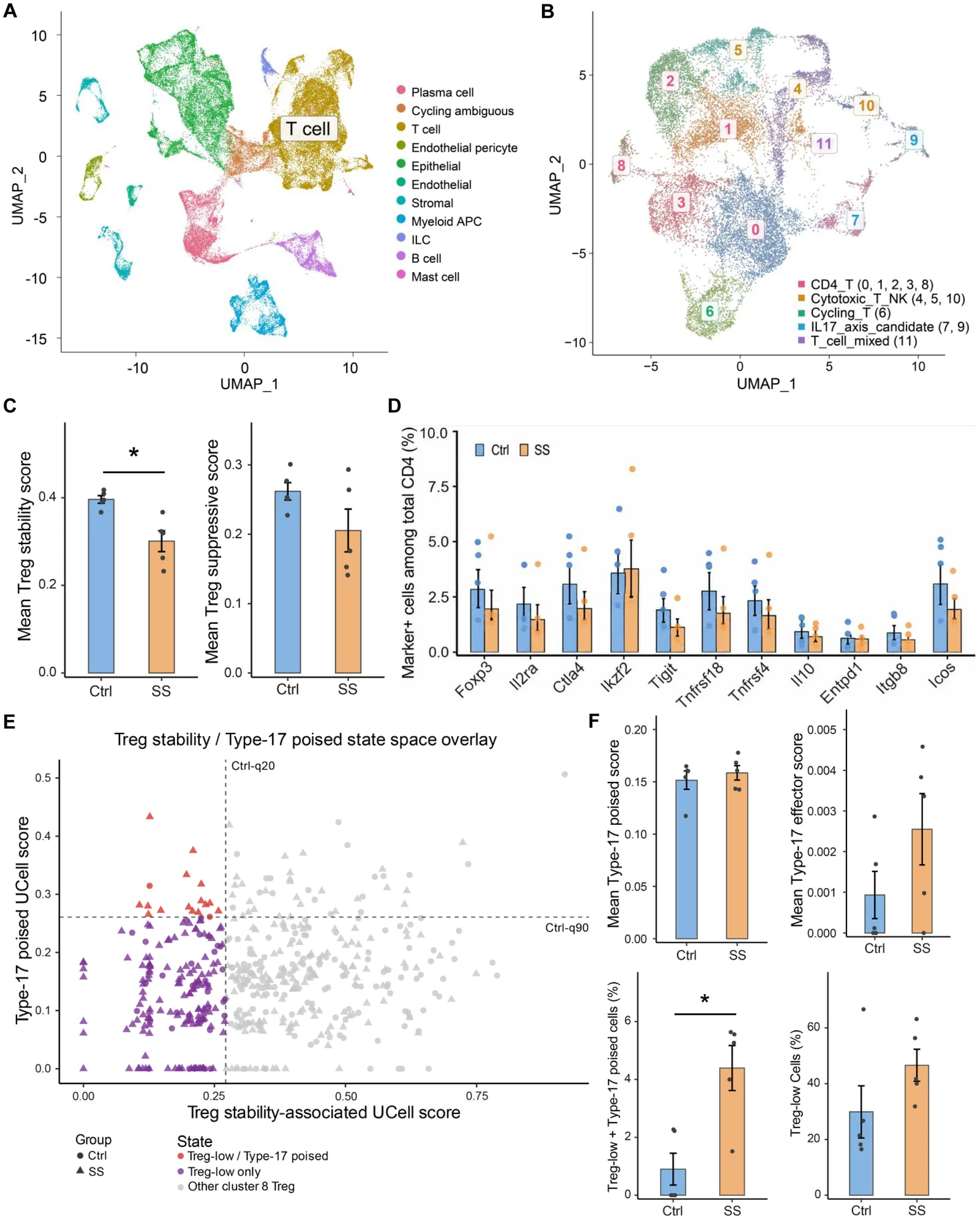
Single-cell transcriptomic profiling reveals Treg instability and Type-17-associated transcriptional features following *Streptococcus salivarius* administration. **(A)** Dimensionality reduction and annotation of major immune cell populations identified by single-cell RNA sequencing of colonic immune cells. **(B)** Sub-clustering of T cells into 12 transcriptionally distinct populations, with cluster 8 annotated as regulatory T cells (Tregs). **(C)** UCell module scores reflecting Treg stability and suppressive function in control and *S. salivarius*-colonized mice. Both transcriptional programs were reduced following *S. salivarius* colonization. **(D)** Distribution of classical Treg-associated markers among CD4^+^ T cells. **(E)** State-space analysis integrating Treg stability and Type-17-poised scores. Dashed thresholds indicate the predefined q20 threshold for Treg stability and q90 threshold for the Type-17-poised state. **(F)** Sample-level quantification of Treg-low/Type-17-poised cells, Treg-low cells, and Type-17-associated transcriptional scores within cluster 8. *S. salivarius* colonization increased the frequency of Treg-low/Type-17-poised cells. Sample-level statistical comparisons were performed using two-sided Mann–Whitney U tests, with individual mice treated as biological replicates. *P < 0.05.

Module scoring analysis revealed that *S. salivarius* exposure significantly altered Treg-associated transcriptional programs. *S. salivarius*-associated Tregs exhibited a significantly lower Treg-stability-associated score (**Figure 2C**, *P* < 0.05), whereas the suppressive-program score showed a downward trend. Consistently, the proportions of cells expressing classical Treg-associated markers were altered among total CD4^+^ T cells, consistent with altered regulatory T-cell transcriptional states (**Figure 2D**).

Given the functional plasticity of Tregs, we further examined whether *S. salivarius* exposure was associated with acquisition of Type-17-associated features. State-space analysis based on Treg stability and Type-17-poised scores demonstrated that a greater proportion of SS-derived Tregs exhibited lower Treg stability-associated transcriptional scores and increased Type-17-associated transcriptional signatures (**Figure 2E**). Quantitative analysis confirmed the frequency of Treg-low/Type-17-poised cells was significantly increased in *S. salivarius* exposure mice (**Figure 2F**, *P* < 0.05), whereas Type-17 effector scores were numerically higher. These findings indicate that *S. salivarius* reshapes Treg transcriptional states toward a profile characterized by reduced stability-associated and increased Type-17-associated signatures.

To determine whether these alterations extended beyond the predefined Treg stability and Type-17-associated gene programs, we performed an additional transcriptome-wide sample-level pseudobulk analysis of cluster 8 Tregs. No individual gene reached statistical significance after correction for multiple testing (BH FDR < 0.05), indicating that the available dataset did not support statistically significant gene-level differential expression after transcriptome-wide correction. We therefore avoided assigning mechanistic significance to individual genes and instead examined coordinated transcriptional changes at the pathway level using the complete ranked gene list.

Unbiased preranked GSEA identified 77 GO Biological Process terms and 15 Reactome pathways that were significantly enriched at BH FDR < 0.05 after multiple-testing correction within each gene-set collection. The top 20 pathways from each collection are shown in **Supplementary Figure S1**. *S. salivarius*-associated Tregs exhibited preferential enrichment of mitochondrial respiration and energy-production programs, including oxidative phosphorylation, ATP synthesis-coupled electron transport, the electron transport chain, and aerobic respiration. Reactome analysis similarly identified enrichment of respiratory electron transport and mitochondrial complex biogenesis, together with RNA-processing and translation-related pathways, in *S. salivarius*-associated Tregs. In contrast, control Tregs showed preferential enrichment of pathways related to T-cell and lymphocyte activation, cell–cell adhesion, regulation of immune effector processes, leukocyte-mediated immunity, and MHC class II antigen presentation (**Supplementary Figure S1**).

Together, these unbiased transcriptome-wide analyses demonstrate coordinated pathway-level remodeling of the Treg compartment following *S. salivarius* exposure and extend the predefined signature-based findings by identifying broader pathway-level differences involving cellular metabolic and immune-regulatory processes.

### 3.3 *S. salivarius* shifts intestinal Th17/Treg homeostasis toward a Th17-biased state

Flow-cytometric analysis of colonic lamina propria mononuclear cells (LPMCs) was performed to evaluate the effects of *S. salivarius* administration on CD4^+^ T-cell subsets. Following sequential gating of live CD45^+^ leukocytes and CD4^+^ T cells, intracellular staining revealed a significantly higher frequency of IL-17A^+^ Th17 cells in the *S. salivarius*-treated group than in the corresponding control group in both ABX and SPF mice (**Figures 3B, C**). In contrast, the frequency of Foxp3^+^ Treg cells showed a downward trend in ABX mice and was significantly reduced in SPF mice, resulting in a shift toward a Th17-biased immune phenotype.

**Figure 3:**
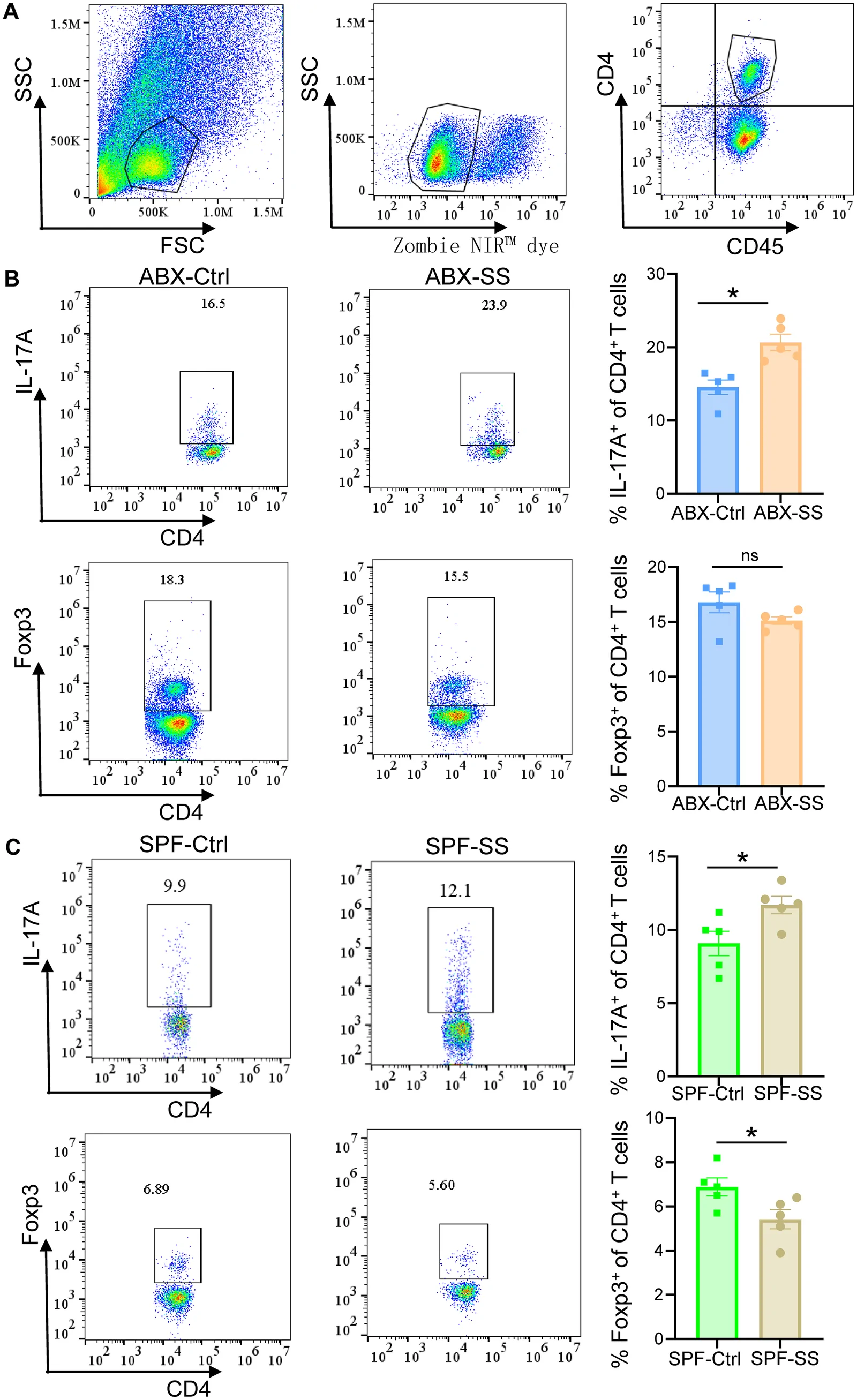
*Streptococcus salivarius* disrupts intestinal Th17/Treg immune homeostasis. **(A)** Representative flow-cytometric gating strategy used to identify live CD45^+^CD4^+^ T cells, IL-17A^+^ Th17 cells, and Foxp3^+^ regulatory T cells (Tregs) in colonic lamina propria mononuclear cells (LPMCs). **(B)** Frequencies of IL-17A^+^ Th17 cells and Foxp3^+^ Tregs among CD4^+^ T cells in ABX-Ctrl and ABX-SS mice. *S. salivarius* administration increased Th17 frequencies, whereas Treg frequencies showed a nonsignificant downward trend. **(C)** Frequencies of Th17 and Treg populations in conventional SPF-Ctrl and SPF-SS mice. SPF-SS mice exhibited increased Th17 frequencies and reduced Treg frequencies compared with SPF-Ctrl mice. Data are presented as mean ± SEM. Comparisons between corresponding control and *S. salivarius*-treated groups were performed using two-sided unpaired Student’s t-tests. *P < 0.05.

Collectively, these findings demonstrate that the transcriptional changes identified by scRNA-seq are accompanied by measurable alterations in CD4^+^ T-cell composition, indicating that *S. salivarius* promotes Th17-biased immune polarization in the intestinal lamina propria. To determine whether these immune alterations depended on the intestinal microbial background, we further performed two-way ANOVA using experimental background (ABX vs. SPF) and bacterial treatment (Ctrl vs. SS) as independent factors. The analysis demonstrated a strong main effect of experimental background on both Th17 and Treg frequencies, whereas only a marginal microbiota × treatment interaction was observed for Th17 cells and no interaction was detected for Treg cells (**Supplementary Figures 2C, D**). These findings indicate that *S. salivarius* induced a quantitively similar shift toward Th17-biased immunity under both microbial conditions, although baseline immune composition differed between microbiota-depleted and conventional mice.

### 3.4 Gut-associated Th17 immune alterations extend to mesenteric lymph nodes

To determine whether the immune alterations observed in the intestinal mucosa extended to regional lymphoid tissues, CD4^+^ T-cell subsets were further analyzed in MLNs. Using the same sequential flow-cytometric gating strategy, *S. salivarius* administration was associated with a significantly increased frequency of IL-17A^+^ Th17 cells among CD4^+^ T cells in both ABX and SPF mice (**Figures 4B, C**). The frequency of Foxp3^+^ Treg cells remained unchanged in ABX mice and showed a nonsignificant downward trend in SPF mice.

**Figure 4:**
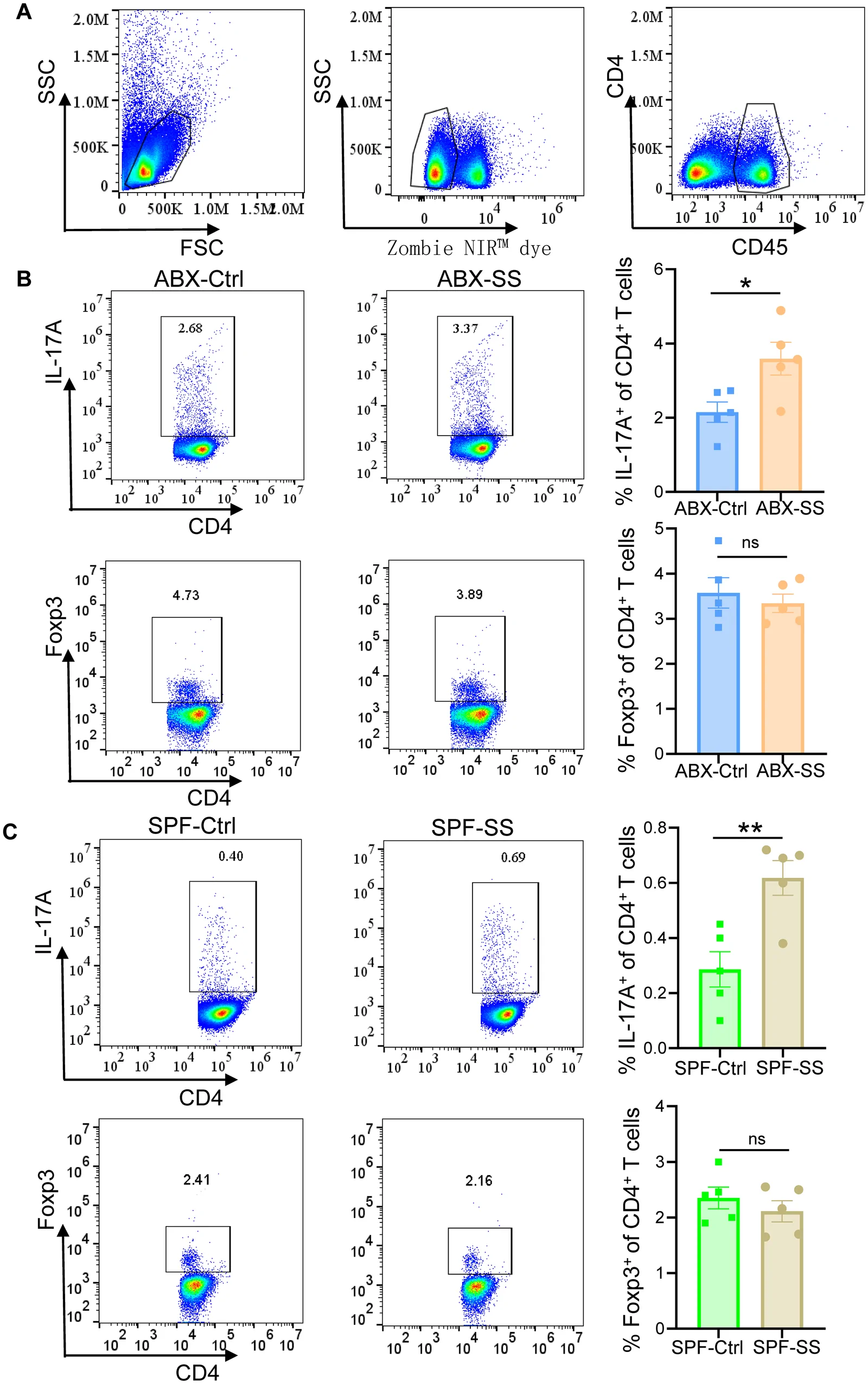
Gut-associated Th17 immune alterations are also observed in mesenteric lymph nodes following *Streptococcus salivarius* administration. **(A)** Representative flow-cytometric gating strategy for live CD45^+^CD4^+^ T cells, IL-17A^+^ Th17 cells, and Foxp3^+^ Tregs in mesenteric lymph nodes (MLNs). **(B)** Frequencies of Th17 and Treg populations in MLNs from ABX-Ctrl and ABX-SS mice. *S. salivarius* administration increased the proportion of IL-17A^+^ Th17 cells, whereas Treg frequencies were unchanged. **(C)** Frequencies of Th17 and Treg populations in MLNs from SPF-Ctrl and SPF-SS mice. SPF-SS mice exhibited a pronounced increase in IL-17A^+^ Th17 cells, accompanied by a nonsignificant downward trend in Foxp3^+^ Tregs. Data are presented as mean ± SEM. Comparisons between corresponding control and *S. salivarius*-treated groups were performed using two-sided unpaired Student’s t-tests. *P < 0.05; **P < 0.01.

Similar factorial analyses were performed for MLN immune populations. Consistent with the colonic findings, experimental background significantly influenced both Th17 and Treg frequencies, whereas only a marginal microbiota × treatment interaction was detected for Th17 cells and no interaction was observed for Treg cells (**Supplementary Figures 2E, F**).

### 3.5 *S. salivarius* is associated with an IL-17A-dominated inflammatory response

To determine whether the observed Th17-biased immune polarization was accompanied by functional inflammatory responses, cytokine levels were quantified in colon tissues and peripheral serum.

In ABX mice, *S. salivarius* administration significantly increased IL-17A levels in colon tissues (**Figure 5A**, *P* < 0.05) and serum (**Figure 5B**, *P* < 0.01). Similar changes were observed in SPF mice, in which colonic IL-17A levels were significantly elevated following *S. salivarius* administration (**Figure 5C**, *P* < 0.05), whereas serum IL-17A showed an increasing trend that did not reach statistical significance (**Figure 5D**). In contrast, the concentrations of IL-6, IL-1β, IL-10, and TGF-β remained comparable between groups in both colon tissues and serum.

**Figure 5:**
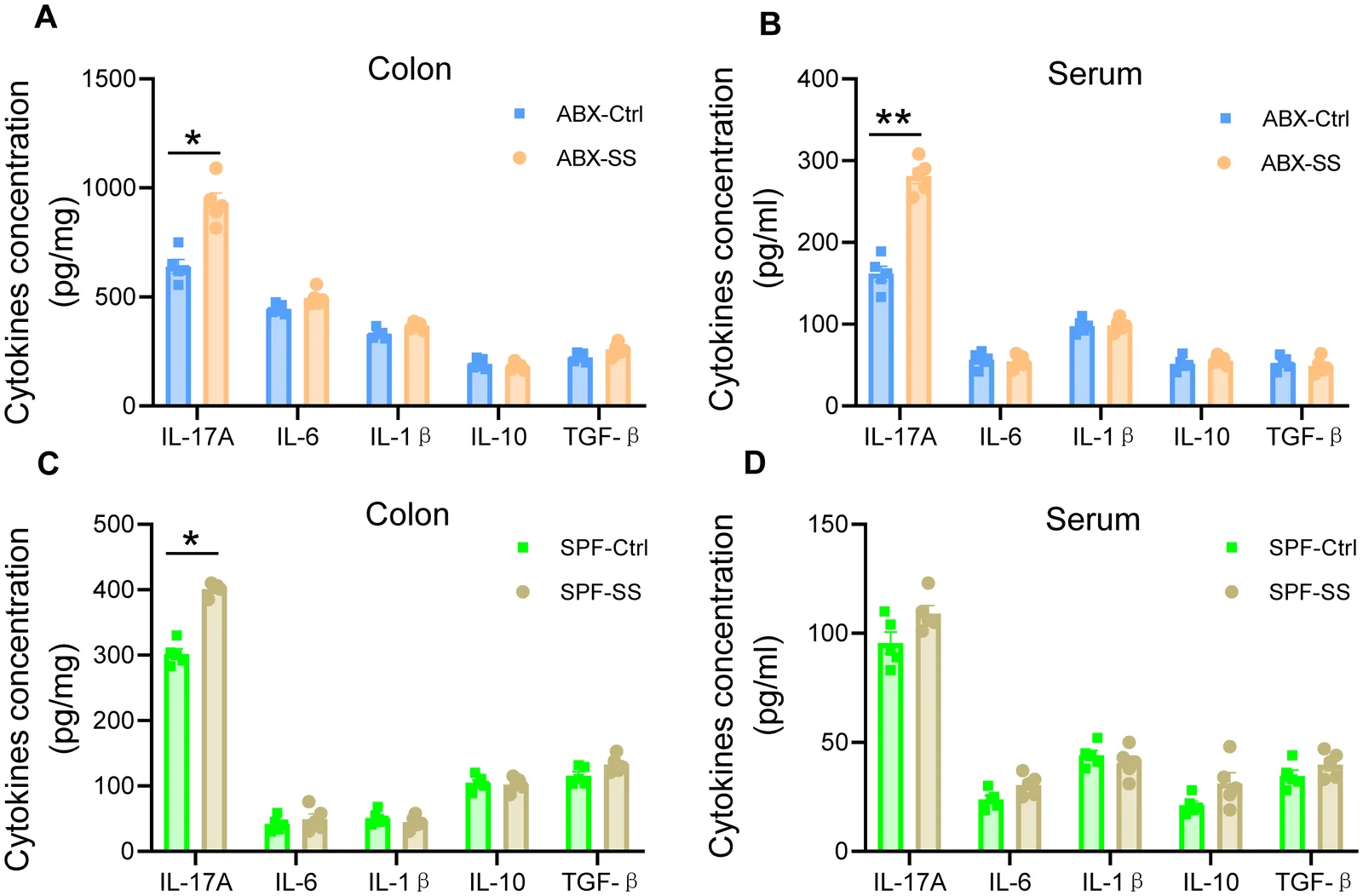
*Streptococcus salivarius* establishes an IL-17A-dominated intestinal and systemic inflammatory signature. **(A)** Cytokine levels in colon tissues from ABX-Ctrl and ABX-SS mice. *S. salivarius* administration significantly increased IL-17A levels. **(B)** Serum cytokine levels in ABX mice. Serum IL-17A was significantly elevated in ABX-SS mice compared with ABX-Ctrl mice. **(C)** Cytokine levels in colon tissues from SPF-Ctrl and SPF-SS mice. Colonic IL-17A was increased following *S. salivarius* administration. **(D)** Serum cytokine levels in SPF mice. IL-17A showed an upward trend in SPF-SS mice but did not reach statistical significance. Across both experimental models, IL-6, IL-1β, IL-10, and TGF-β showed no significant differences between *S. salivarius*-treated and corresponding control mice. Data are presented as mean ± SEM. Comparisons between corresponding control and *S. salivarius*-treated groups were performed using two-sided unpaired Student’s t-tests. *P < 0.05; **P < 0.01.

Collectively, these findings demonstrate that the cellular immune remodeling induced by *S. salivarius* is accompanied by a selective IL-17A elevation in colonic tissues, accompanied by systemic IL-17A elevation that was significant in ABX mice and showed a similar upward trend in SPF mice. Two-way ANOVA further demonstrated that experimental background significantly affected both colonic and serum IL-17A concentrations. Importantly, significant microbiota × treatment interactions were identified for both tissues, indicating that although *S. salivarius* was associated with increased IL-17A production in both experimental models, the magnitude of this inflammatory response differed according to microbial background (**Supplementary Figures 2A, B**).

## 4 Discussion

The gut–brain axis has emerged as an important framework for understanding how microbial dysbiosis contributes to neuropsychiatric disorders, including SCZ, yet the specific microbial species and immune responses involved remain poorly defined (1–5, 16). In the present study, we show that intestinal enrichment of a representative SCZ-associated *S. salivarius* with coordinated remodeling of intestinal adaptive immunity. This remodeling was characterized by altered Treg-associated transcriptional programs, increased Type-17-associated transcriptional features, Th17-biased CD4^+^ T-cell response, and selective elevation of IL-17A production. These findings identify an intestinal immune response associated with exposure to an SCZ-associated oral-origin bacterium. Because the present study focused on intestinal and peripheral immune alternations, the results should be interpreted as defining one immunological component potentially relevant to the oral–gut–brain axis rather than establishing a causal pathway to SCZ (1, 3, 16).

A major strength of this study is the integration of complementary experimental approaches across transcriptional, cellular and cytokine levels. Single-cell transcriptomic analyses revealed lower Treg stability-associated transcriptional scores together with increased Type-17-associated features following *S. salivarius* exposure. Rather than demonstrating functional loss of Treg activity directly, these findings indicate a shift in Treg-associated transcriptional state. This interpretation is consistent with the well-established functional plasticity of CD4^+^ T-cell subsets and the capacity of regulatory and Type-17-associated programs to undergo context-dependent remodeling under inflammatory conditions (14, 15, 24). Flow-cytometric analyses further demonstrated increased frequencies of IL-17A^+^ CD4^+^ T cells together with changes in Foxp3^+^ Treg frequencies in intestinal tissues, while cytokine measurements showed selective elevation of IL-17A. The convergence of these independent readouts supports coordinated adaptive immune remodeling rather than an isolated alteration in a single immune marker.

Transcriptome-wide pseudobulk analysis did not identify individual genes that remained statistically significant after correction for multiple testing. This finding argues against assigning mechanistic importance to individual transcripts in the present dataset. However, preranked GSEA identified significant pathway-level differences between control and *S. salivarius*-exposed Tregs. Pathways related to mitochondrial respiration, oxidative phosphorylation, electron transport, RNA processing, and translation were preferentially enriched in SS-associated Tregs, whereas pathways associated with lymphocyte activation, immune-effector regulation, cell adhesion, and antigen presentation were preferentially enriched in controls. These pathway-level findings complement the predefined UCell analyses and suggest that *S. salivarius*-associated Treg remodeling involves coordinated changes in metabolic and immune-regulatory programs. The absence of transcriptome-wide significant individual genes may reflect relatively modest transcriptional differences together with the limited number of biological replicates, and these pathway-level observations therefore require validation in larger and independent datasets.

The present findings extend previous microbiome studies in SCZ by providing experimental evidence that an SCZ-associated oral-origin bacterial isolate can influence intestinal CD4^+^ T-cell homeostasis after repeated oral exposure. Previous studies have consistently reported alterations in gut microbial composition in patients with SCZ, although most have primarily been observational or associative in nature (1–5). Experimental work has also demonstrated that gut microbiota derived from patients with SCZ can influence metabolic pathways and SCZ-relevant behavioral phenotypes in recipient mice (3). Beyond psychiatric disorders, accumulating evidence indicates that oral-derived bacteria can reach the intestinal ecosystem and modify mucosal immune responses under permissive conditions (6, 7, 9). For example, ectopic intestinal colonization by oral bacteria can induce intestinal T-cell responses and inflammation (6), while *Fusobacterium nucleatum* has been shown to modify the local immune microenvironment in colorectal disease (8). Against this background, our findings indicate that intestinal enrichment of a representative SCZ-associated *S. salivarius* isolate is accompanied by increased Th17 responses and changes in Treg-associated transcriptional and cellular features. The novelty of the present study therefore lies not in identifying Th17/Treg imbalance as a new immune phenomenon, but in experimentally linking exposure to an SCZ-associated oral bacterium with adaptive immune remodeling in the intestine.

This immune phenotype may be relevant to SCZ because dysregulation of the Th17/Treg axis and IL-17-related signaling has previously been reported in clinical studies of SCZ. Increased Th17-related cytokine activity, enhanced Th17 responses, and reduced regulatory T-cell populations have been observed in patients with schizophrenia, including drug-naive and first-episode populations (17, 18, 25). These observations are broadly consistent with the immunological perspective that altered adaptive immune responses may contribute to microbiota–gut–brain interactions in SCZ (16). However, the upstream microbial factors that may contribute to these immune alterations remain poorly understood. Our findings indicate that intestinal enrichment of an SCZ-associated oral isolate can generate a partially overlapping immune phenotype in mice. This observation supports the possibility that disease-associated microbial exposures may contribute to immune abnormalities relevant to SCZ, but it does not establish that *S. salivarius* causes SCZ or that the immune alterations observed here occur through the same mechanisms in patients.

The inclusion of both ABX and conventional SPF mice further allowed us to examine the extent to which the surrounding microbial environment influenced the host response to *S. salivarius*. Qualitatively similar increases in Th17-associated responses were observed under both conditions, indicating that the immunological effects of *S. salivarius* were not restricted to a microbiota-depleted background. At the same time, factorial analyses demonstrated that microbial context influenced the magnitude of these responses. Significant microbiota × treatment interactions were observed for colonic and serum IL-17A concentrations, whereas interaction effects for Th17 frequencies were marginal and no significant interaction was detected for Treg frequencies. These findings indicate that *S. salivarius* exposure was associated with a broadly similar Th17-biased immune pattern across the two experimental backgrounds, while the magnitude of downstream cytokine responses depended partly on the resident microbial context. This interpretation is biologically plausible because the intestinal microbiota contributes to colonization resistance, microbial competition, antigen availability, and basal immune tone, all of which can influence host responses to newly introduced microorganisms (6, 7, 9, 23).

Immune alterations were detected across several anatomically connected compartments. Treg-associated transcriptional remodeling and changes in intestinal CD4^+^ T-cell composition were accompanied by increased Th17 frequencies in MLNs and by changes in circulating IL-17A. These observations are consistent with the regional organization of intestinal immunity, in which anatomically distinct draining lymph nodes integrate immune signals from different intestinal regions and contribute to the coordination of mucosal and systemic adaptive immune responses (22, 23). However, because the present study did not directly trace immune-cell migration or establish temporal causality between these compartments, the findings should not be interpreted as demonstrating a sequential mucosa-to-lymph-node-to-systemic pathway.

IL-17A is of particular interest in the context of neuroimmune communication. Th17-associated responses can influence inflammatory signaling within the central nervous system, and experimental studies have shown that IL-17-related signaling can contribute to neuronal dysfunction and disruption of blood–brain barrier properties under inflammatory conditions (20, 21). More broadly, adaptive immune cells have complex bidirectional interactions with the central nervous system and can influence neuroimmune homeostasis (26). In the present study, IL-17A was significantly increased in colonic tissues in both experimental models and in serum from antibiotic-pretreated mice, whereas serum IL-17A showed a nonsignificant upward trend in conventional SPF mice. These findings raise the possibility that intestinal immune remodeling associated with *S. salivarius* exposure may influence broader host immune responses relevant to microbiome–brain interactions. Nevertheless, behavioral phenotypes, blood–brain barrier integrity, neuroinflammation, and central nervous system immune changes were not assessed. Accordingly, the present study defines an intestinal immune response potentially relevant to the oral–gut–brain axis rather than a direct mechanism linking *S. salivarius* to SCZ (16, 20, 21, 26).

## 5 Conclusions

In summary, this study demonstrates that intestinal enrichment of a representative SCZ-associated oral-origin *S. salivarius* isolate following repeated oral administration is associated with coordinated remodeling of intestinal adaptive immunity. These changes include altered Treg-associated transcriptional programs, pathway-level differences in metabolic and immune-regulatory processes, increased Type-17-associated transcriptional features, Th17-biased CD4^+^ T-cell responses, and selective elevation of IL-17A. Together, these findings provide experimental evidence linking exposure to an SCZ-associated oral bacterium with intestinal immune remodeling and support the broader concept that microbial–immune interactions may represent one component of the microbiota–gut–brain framework in SCZ (1, 16). However, the present findings do not establish long-term intestinal colonization, a strain-specific effect, or a causal relationship between *S. salivarius* and SCZ. Further studies incorporating comparative strain analyses, longitudinal assessment of bacterial persistence, direct functional evaluation of Treg activity, mechanistic interrogation of host–microbial interactions, absolute immune-cell quantification, and behavioral or neuroimmune assessments will be required to clarify the contribution of oral-derived bacteria to microbiome–brain interactions in SCZ.

## Data Availability

The datasets presented in this study can be found in online repositories. The names of the repository/repositories and accession number(s) can be found below: <b> https://ngdc.cncb.ac.cn/, PRJCA071267.

## Supplementary material

Supplementary Figure 1 Transcriptome-wide gene set enrichment analysis of cluster 8 Treg cells. Preranked gene set enrichment analysis (GSEA) was performed using the complete edgeR-ranked gene list without applying a gene-level significance cutoff. **(A)** Top Gene Ontology (GO) Biological Process terms and **(B)** top Reactome pathways, ranked by Benjamini–Hochberg (BH)-adjusted false discovery rate (FDR) followed by the absolute normalized enrichment score (|NES|). Positive NES values indicate enrichment toward the *S. salivarius* (SS) group, whereas negative NES values indicate enrichment in the control (Ctrl) group. Point size represents −log10 (BH-adjusted FDR), and filled circles denote significantly enriched pathways points denote pathways (BH FDR < 0.05). Compared with Ctrl Treg cells, SS-associated Tregs were preferential enriched for pathways related to mitochondrial respiration, oxidative phosphorylation, electron transport, and ATP synthesis, whereas immune-cell activation, leukocyte and lymphocyte activation, cell adhesion, antigen presentation and immune system processes. These results indicate a shift from immune activation- and cell adhesion-related transcriptional programs toward mitochondrial respiratory and oxidative metabolic pathways in SS-associated Treg cells.

Supplementary Figure 2 Two-way ANOVA analysis of the effects of intestinal microbiota status and *Streptococcus salivarius* administration on Th17/Treg immune responses and IL-17A production. Two-way ANOVA was performed to evaluate the effects of intestinal microbiota status (SPF versus ABX) and *S. salivarius* administration (Ctrl versus SS), as well as their interaction, on immune phenotypes. Four experimental groups were analyzed: SPF-Ctrl, SPF-SS, ABX-Ctrl, and ABX-SS. **(A)** Colonic IL-17A concentrations. **(B)** Serum IL-17A concentrations. **(C)** Frequencies of IL-17A^+^ Th17 cells among CD4^+^ T cells in LPMCs. **(D)** Frequencies of Foxp3^+^ Treg cells among CD4^+^ T cells in LPMCs. **(E)** Frequencies of IL-17A^+^ Th17 cells among CD4^+^ T cells in MLNs. **(F)** Frequencies of Foxp3^+^ Treg cells among CD4^+^ T cells in MLNs. *P* values for the main effects of microbiota status, *S. salivarius* treatment, and their interaction are shown above each panel. Different lowercase letters indicate statistically significant differences among the four groups based on Tukey’s multiple-comparison test following two-way ANOVA. Data are presented as box-and-whisker plots showing the median, interquartile range, and minimum-to-maximum values, with individual mice represented by dots.
