# Supplementary material for: An oral-origin Streptococcus salivarius associated with schizophrenia reshapes intestinal T-cell immunity and promotes IL-17A-dominated inflammation
Source: Front Immunol. 2026 Oct 15;17:1978713. doi: 10.3389/fimmu.2026.1978713 (PMC13716591; doi:10.3389/fimmu.2026.1978713)
Supplement: Supplementary file 1 [file Table1.docx]

**Supplementary Table 1. Primers used for qPCR**

| **Primers** | **Sequence (5’-3')** |
| --- | --- |
| *S. Salivarius*-F | GGGAAGCATTATGGATTACC |
| *S. salivarius*-R | ACACATCAAGGACTGACTTATC |
